# Assessing the clinical benefit from medical therapies in CNS malignancies: Refining the ESMO Magnitude of Clinical Benefit Scale and the ASCO Value Framework

**DOI:** 10.1093/noajnl/vdaf127

**Published:** 2025-06-20

**Authors:** Sylvia C Kurz, Mirjam Renovanz, Michael Bitzer, Maximilian Niyazi, Nisar Malek, Marcos Tatagiba, Ghazaleh Tabatabai

**Affiliations:** Center for Personalized Medicine Tübingen, University Hospital Tübingen, Eberhard Karls University Tübingen, Tübingen, Germany; Center for Neuro-Oncology, Comprehensive Cancer Center Tübingen-Stuttgart, University Hospital Tübingen, Eberhard Karls University Tübingen, Tübingen, Germany; Department of Neurology & Interdisciplinary Neuro-Oncology, University Hospital Tübingen, Hertie Institute for Clinical Brain Research, Eberhard Karls University Tübingen, Tübingen, Germany; Department of Neurosurgery, University Hospital Tübingen, Eberhard Karls University Tübingen, Tübingen, Germany; Center for Personalized Medicine Tübingen, University Hospital Tübingen, Eberhard Karls University Tübingen, Tübingen, Germany; Center for Neuro-Oncology, Comprehensive Cancer Center Tübingen-Stuttgart, University Hospital Tübingen, Eberhard Karls University Tübingen, Tübingen, Germany; Department of Neurology & Interdisciplinary Neuro-Oncology, University Hospital Tübingen, Hertie Institute for Clinical Brain Research, Eberhard Karls University Tübingen, Tübingen, Germany; Department of Internal Medicine I, University Hospital Tübingen, Eberhard Karls University Tübingen, Tübingen, Germany; Center for Personalized Medicine Tübingen, University Hospital Tübingen, Eberhard Karls University Tübingen, Tübingen, Germany; Department of Radiation Oncology, University Hospital Tübingen, Tübingen, Germany; Center for Personalized Medicine Tübingen, University Hospital Tübingen, Eberhard Karls University Tübingen, Tübingen, Germany; Center for Neuro-Oncology, Comprehensive Cancer Center Tübingen-Stuttgart, University Hospital Tübingen, Eberhard Karls University Tübingen, Tübingen, Germany; Department of Internal Medicine I, University Hospital Tübingen, Eberhard Karls University Tübingen, Tübingen, Germany; Center for Personalized Medicine Tübingen, University Hospital Tübingen, Eberhard Karls University Tübingen, Tübingen, Germany; Department of Neurosurgery, University Hospital Tübingen, Eberhard Karls University Tübingen, Tübingen, Germany; Center for Personalized Medicine Tübingen, University Hospital Tübingen, Eberhard Karls University Tübingen, Tübingen, Germany; Center for Neuro-Oncology, Comprehensive Cancer Center Tübingen-Stuttgart, University Hospital Tübingen, Eberhard Karls University Tübingen, Tübingen, Germany; Center for Personalized Medicine Tübingen, University Hospital Tübingen, Eberhard Karls University Tübingen, Tübingen, Germany; Center for Neuro-Oncology, Comprehensive Cancer Center Tübingen-Stuttgart, University Hospital Tübingen, Eberhard Karls University Tübingen, Tübingen, Germany; Department of Neurology & Interdisciplinary Neuro-Oncology, University Hospital Tübingen, Hertie Institute for Clinical Brain Research, Eberhard Karls University Tübingen, Tübingen, Germany

**Keywords:** magnitude of clinical benefit, outcome assessment, quality of life, targeted therapy, toxicity

## Abstract

**Background:**

An objective assessment of patient-centered clinical benefit derived from an anticancer therapy weighs clinical and radiographic outcome against treatment-related burden. Both, the European Society for Medical Oncology (ESMO) and the American Society of Clinical Oncology (ASCO) have developed assessment tools to guide such evaluations. These tools, however, have not been validated for application to neuro-oncology studies.

**Methods:**

We applied the ESMO-Magnitude of Clinical Benefit (ESMO-MCBS) v1.1. and ASCO net health benefit v2 scales to 16 neuro-oncological studies. We developed the Neuro-Oncology Magnitude of Clinical Benefit (Neuro-MCBS) scale and tested its application in select patient cohorts of MTB@ZPM (NCT03503149), a longitudinal observational study that provides comprehensive molecular profiling, consensus-guided and ranked targeted therapy recommendations and prospective assessment of clinical outcome.

**Results:**

Of 10 randomized phase 3 clinical trials and 6 single-arm clinical studies, 9 and 5 were scoreable by ESMO-MCBS v1.1, respectively. A score of 4 was achieved by 5 of the phase 3 and one of the single-arm studies, signifying clinical benefit. Application to these neuro-oncology studies was limited by high variability in criteria used to assess treatment response and lack of credit to therapies that lead to disease control. Based hereon, we developed the Neuro-MCBS suitable to specific needs of neuro-oncology studies and found it to be practicable and applicable to these landmark neuro-oncology studies and various treatment cohorts in MTB@ZPM.

**Conclusions:**

The Neuro-MCBS is a comprehensive, clinically relevant tool and particularly suitable to assess the clinical benefit derived from novel antitumor therapies in neuro-oncology studies.

**Key Points:**

- The Neuro-Magnitude of Clinical Benefit Scale was used to evaluate 16 neuro-oncology studies.
- The net clinical benefit derived from neuro-oncological therapies varied greatly.
- Reporting practices are variable and limit objective comparability.

**Importance of the Study**

In a healthcare environment in which there is an ever so rapid expansion of novel anticancer therapies, an assessment of the clinical benefit from any given therapy needs to weigh the expected outcome against treatment-related burden. Here, we applied the ESMO-Magnitude of Clinical Benefit Scale (ESMO-MCBS) v1.1 and the ASCO Health Value Framework v2 to landmark clinical neuro-oncology studies and observed notable variations in perceived net clinical benefit derived from common medical therapies used in neuro-oncology. Application of these scales was partially limited due to variable assessment criteria being used and limited reporting of toxicity and quality-of-life data. Based hereon, the Neuro-Oncology Magnitude of Clinical Benefit Scale (Neuro-MCBS) was developed as an assessment tool more suitable to neuro-oncology studies. The Neuro-MCBS represents a comprehensive and practicable tool that can help clinicians to assess the magnitude of clinical benefit derived from medical therapies used in neuro-oncology.

Assessment of clinical benefit (“will this therapy be worth it?”) of any anticancer therapy comprises an evaluation of the expected magnitude of improvement in clinically relevant outcome parameters weighed against the burden of treatment, i.e. side effects but also financial, logistical, and administrative features associated with the treatment.^1^ In a healthcare environment, in which there is an ever so rapid expansion of novel and costly therapies, imaging and other healthcare technologies, an evidence-based and value-oriented assessment of the clinical benefit derived from a therapy becomes increasingly important for patients, physicians and other stakeholders in the field.^2,3^

The last 2 decades have been marked by significant progress in understanding of the biological and molecular underpinnings of various Central Nervous System (CNS) tumors. However, this has translated into only few therapeutic breakthroughs for neuro-oncological indications.^4^ For patients with the most common malignant brain tumor, glioblastoma, treatment options remain limited, for various reasons.^5^ Once established therapies fail, glioblastoma patients are left with limited or no treatment options. Yet, a majority of glioblastoma patients will receive treatments towards the end of life.^6,7^ With the nowadays widespread availability of detailed molecular tissue analyses using various platforms, targeted therapies are increasingly offered “off label” to patients for whom established therapy options are no longer available or who do not qualify for participation in a clinical trial.^8–10^ While these therapies and interventions might not be ineffective, the magnitude of clinical benefit may be marginal and might come at the cost of side effects and increased logistical and financial burden for patients, their families and physicians.^11,12^

Hence, an objective and valid evaluation of the net clinical benefit of any given medical therapy in the face of potentially associated toxicities is needed. Both, the European Society for Medical Oncology (ESMO) and the American Society of Clinical Oncology (ASCO) independently developed practical tools to provide guidance to physicians, patients and payors when evaluating new therapies.^13,14^ The ESMO-Magnitude of Clinical Benefit Scale (ESMO-MCBS) was designed to assist stakeholders in the oncological community with a structured and statistically validated approach to evaluate clinical research data, i.e. comparative clinical trials in which a novel oncological therapy was demonstrated to be statistically superior.^15^ Based on the feedback received, an amended ESMO-MCBS version 1.1 includes Form 3 which allows the assessment of single-arm studies for “orphan diseases” or diseases with “high unmet need.”^14^ The ASCO net health benefit (ASCO-NHB) score was designed to assist patients and their oncologists in the value-oriented evaluation of oncological therapies based on clinical benefit, toxicity, symptom palliation, and cost. It is applicable to randomized clinical trials only.^13^ A comparative assessment of the shared and unique characteristics of these 2 tools has been reported.^16^

Neither the ESMO-MCBS nor the ASCO-NHB were evaluated for their practicality and application in this setting. Yet, there is a need in the neuro-oncology community for a value-oriented and objective assessment of the magnitude of clinical benefit of a novel therapy considering the associated clinical, financial, and administrative toxicity. This is particularly relevant because the feasibility of a therapeutic approach does not necessarily translate into a clinical benefit for patients.

In the present study, we therefore applied the ESMO-MCBS v1.1 and the ASCO-NHB v2 to 10 randomized phase 3 clinical trials in neuro-oncology with demonstrated superiority of the evaluated therapy. We also applied Form 3 of the ESMO-MCBS v1.1 to 6 single-arm clinical studies and propose a modified version, the Neuro-Oncology Magnitude of Clinical Benefit scale (Neuro-MCBS) to address specific needs in neuro-oncology. The Neuro-MCBS was tested for its feasibility and validity in neuro-oncology patient cohorts enrolled in MTB@ZPM, a prospective single-center single-arm clinical study.^8^

## Materials and Methods

### Selection of Clinical Studies

Relevant clinical studies evaluating drug therapies for neuro-oncological conditions were selected based on the references cited in the European Society for Neuro-Oncology (ESMO) Guidelines for Neuro-Oncology,^17^ EANO guidelines on the diagnosis and treatment of diffuse gliomas of adulthood,^4^ and National Comprehensive Cancer Network® (NCCN®) treatment guidelines for CNS Cancers.^18^

### ESMO-Magnitude of Clinical Benefit Scale v1.1

The methodology underlying the ESMO-MCBS v1.1 Forms 1, 2a-c, and 3 is detailed elsewhere^14,15,19^ and can be found online (ESMO-MCBS for Solid Tumours). Form 1 is used to evaluate benefit from adjuvant or neoadjuvant therapies used with curative intent.^15^ Given that the neuro-oncological therapies evaluated here are not likely to be curative, form 1 was deemed not applicable and not used. Form 2a is used to evaluate comparative clinical studies that used overall survival (OS) as the primary study endpoint and that demonstrated statistical superiority of the tested therapy. Based on the lower limit of the 95% confidence interval (LL95%CI) of the hazard ratio (HR) and the OS gain, a preliminary score on a 4-point scale is rendered. The preliminary score can be upgraded by 1 point for a maximal score of 5, if objective quality of life (QoL) assessments shows significant improvement and/or if there is a significant reduction in toxicity compared to the standard therapy arm. Form 2b is used for comparative studies where PFS is used as the primary endpoint. A preliminary score based on HR LL95%CI and PFS gain can be up- or downgraded based on specific criteria (early stopping/crossover, toxicity and QoL assessments). Form 2c applies to comparative studies with primary endpoints other than OS or PFS, or to non-inferiority studies. Assessments are based on response rate (RR), toxicity and QoL. Form 3 is applied to single-arm studies in “orphan diseases” or areas of “high unmet need.” Preliminary scoring on a 3-point scale is based on PFS, objective response rate (ORR), and duration of response (DoR). The preliminary score can be upgraded for improvements in QoL and/or toxicity. Forms 2b, 2c, and 3 allow for a maximal score of 4. For all forms, a final score of 4 or 5 indicates high-level of clinical benefit.

### ASCO Value Framework NHB Score v2

The ASCO reported value frameworks for the “adjuvant setting” and for “advanced disease.”^13^ Based on the value framework for “advanced disease,” a net health benefit (NHB) score is rendered based on assessment of the available clinical trial data (HR, OS, PFS, or RR), toxicity and “bonus points.” A maximum score of 130 can be achieved. The value framework for “adjuvant setting” is based on an assessment of clinical trial outcome parameters and toxicity only for a maximum score of 100.

### MTB@ZPM Design

The Molecular Tumor Board (MTB) Tübingen at the Center for Personalized Medicine Tübingen (MTB@ZPM, NCT03503149) is a prospective single-center single-arm clinical study that aims at molecular-guided stratification and therapy of patients with advanced tumor diseases, continuously recruiting since March 2018. The MTB@ZPM workflow provides comprehensive molecular profiling, consensus-guided and ranked targeted therapy recommendations and collection of prospective clinical outcome data. The study patient cohorts investigated here comprised of adult patients (≥ 18 years old) with tumors affecting the CNS who received targeted therapies based on the results of our comprehensive molecular profiling platform and treatment recommendations issued by the institutional MTB.^8^

## Results

### Application of the ESMO-MCBS 1.1 Forms 2a-c and the ASCO value framework for “adjuvant disease” to randomized clinical trials evaluating therapies for CNS malignancies

Ten randomized phase 3 clinical trials were evaluated (Table 1).^20–29^ Nine studies evaluated systemic therapies. One clinical trial evaluating a localized therapy, tumor-treating fields (TTF), was included because the study’s design and outcome measurements were considered in line with current standards and deemed scoreable.^21^ A final ESMO-MCBS score could be assigned to all but 1 study. Of these, 5 studies (55%) scored 4 out of 5 points, signifying high-level of clinical benefit.^22,27–29^ One clinical study with non-inferiority design could not get a score because, per Form 2c of the ESMO-MCBS, assessment of both, significant reduction of toxicity *and* evidence of statistical non-inferiority would have been required. While the study met its non-inferiority primary endpoint, there was insufficient reporting of toxicity, hence a score could not be rendered.^23^ One 3-arm study evaluated hypofractionated radiotherapy and temozolomide, each compared against standard radiotherapy. Since only the temozolomide arm reached statistically significant superiority, an ESMO-MCBS score was determined by assessment of that study arm only.^24^ Another study evaluated the benefit of combining radiation with procarbazine-CCNU-vincristine polychemotherapy in patients with anaplastic oligodendrogliomas. Statistical superiority of combined treatment modality was only reached in a post hoc re-evaluation of clinical outcomes based on underlying molecular phenotypes. We included this study because it was deemed of significant importance to the field, having led to change in treatment recommendations and practice pattens.^26^

**Table 1.** ESMO MCBS 1.1 and ASCO-NHB v2 Assessment of Randomized Phase 3 Clinical Trials for CNS Malignancies

Treatment indication/disease	Study name [Reference]	Intervention vs. Control	No of subjects	1° endpoint	mPFS gain	HR (PFS), point estimate (95% CI)	mOS gain	HR (OS), point estimate (95% CI)	ESMO-MCBS form used	ESMO-MCBS score	ASCO-NHB Score^a^
Subgroup, if applicable											
Glioblastoma, newly diagnosed	EORTC 26981-22981/NCIC CE3 [20]	RT+TMZ vs. RT alone	573	OS	1.9 months	0.63 (0.53–0.75)	2.5 months	0·63 (0·53–0·75)	2a^b^	2	34.8
Glioblastoma, newly diagnosed	EF-14 [21]	RT+TMZ+TTF vs. RT+TMZ	695	PFS	2.7 months	0.63 (0.52–0.76)	4.9 months	0.63 (0.53–0.76)	2a^e^	3	26
Glioblastoma, newly diagnosed, MGMT methylated	CeTeG/NOA-09 [22]	RT+TMZ+CCNU vs. RT+TMZ	144	OS	(-)	0.99 (0·68–1·46)	16.5 months	0.6 (0.35–1.03)	2a	4	37.75
Anaplastic astrocytoma/glioblastoma, newly diagnosed, >65 years old, KPS ≥ 60	NOA-08 [23]	TMZ vs RT	373	OS	(−1.4 months)	1.15 (0.92–1.43)	(−1 month)	1.09 (0.84–1.42)	2c^d^	n/a^f^	n/a^g^
Glioblastoma, newly diagnosed, >60 years old	Nordic Trial [24]	TMZ vs. RT and HFRT vs RT	291	OS							
RT			100								
HFRT			98		(-)	(-)	1.5 months	0.85 (0.64–1.12)	2a^b^	n/a^h^	n/a^h^
TMZ			93		(-)	(-)	2.3 months	0.70 (0.52–0.93)	2a^b^	2	17.1
Glioblastoma, newly diagnosed, >65 years old	Short-course radiation plus Temozolomide in the elderly [25]	HFRT+TMZ vs. HFRT	562	OS	1.4	0.50 (0.41–0.60)	1.7 months	0.67 (0.56–0.80)	2a^b^	2	33^i^
Anaplastic oligodendroglioma/oligoastrocytoma, newly diagnosed	RTOG9402 [26]	PCV → RT vs. RT	291	OS	(-)	(-)	(−0.1 years)	0.79 (0.60–1.04)	2a^b^	n/a^h^	n/a^h^
IDH mutant			156				3.7 years	0.59 (0.40–0.86)		4	33.2
1p19q co-del			10				7.3 years	0.59 (0.37–0.95)		4	33.2
IDH mutant and 1p19q co-del			88				7.9 years	0.49 (0.28–0.85)		4	43.2
IDH mutant, not 1p19q co-del			66				2.2 years	0.56 (0.32–0.99)		4	36.2
Not IDH-mutant, not 1p19q co-del			44				(−0.3 years)	0.99 (0.53–1.86)		n/a^h^	n/a^h^
Anaplastic Oligodendroglioma, newly diagnosed	EORTC 26951 [27]	RT → PCV vs. RT	368	OS/PFS	11.1 months	0.66 (0.52–0.83)	11.7 months	0.75 (0.60–0.95)	2a^b^	4	45^i^
Low-grade glioma, resected, no prior RT/chemotherapy	RTOG 9802 [28]	RT → PCV vs. RT	251	OS	6.4 years	0.5 (0.36–0.68)	5.5 years	0.59 (0.42–0.83)	2a^b^	4	11^j^
Low-grade glioma, resected, no prior RT/chemotherapy	INDIGO [29]	Vorasidenib vs. placebo	331	PFS	16.6 months	0.39 (0.27–0.56)	(-)	(-)	2b^c^	4	79.5

Abbreviations: CI = confidence interval; HFRT = hypofractionated RT; HR = hazard ratio; IDH = Isocitrate dehydrogenase; mEFS = median event-free survival; mOS = median overall survival; mPFS = median progression-free survival; PCV = procarbazine; RT = radiotherapy; TMZ = temozolomide; TTF = tumor-treating fields; n/a = not assessable

^a^ASCO value framework for *adjuvant setting* was used.

^b^Form 2a: not curative, 1° endpoint is OS, scale 1–5.

^c^Form 2b: not curative, 1° endpoint is PFS, scale 1–4.

^d^Form 2c: not curative, 1° endpoint other than OS/PFS or equivalence studies, scale 1–4.

^e^Per Form 2b instructions, Form 2a is to be used if OS benefit demonstrated.

^f^Form 2c could not be completed as toxicity data not sufficient.

^g^ASCO value framework does not consider non-inferiority study design.

^h^No statistical significance reached; ^i^limited report of toxicity data.

^j^Bonus points for tail of curve appeared possible, but no sufficient data.

The ASCO value framework for “advanced disease” was applied to the same 10 studies. A NHB score was reached in 9 studies, albeit with variable scores suggesting variable net clinical benefit. One study was not evaluable because ASCO-NHB v2 does not allow the assessment of studies with non-inferiority design.^23^

### Application of the ESMO-MCBS v1.1 Form 3 to single-arm clinical studies of medical therapies for CNS malignancies.

We assessed 6 single-arm phase 1 and 2 studies by using Form 3 of the ESMO-MCBS v1.1 (Table 2).^30–37^ All but 1 study were assessable. Of these, 1 study achieved an ESMO-MCBS score of 4 and 3 studies received a score of 3. While all studies were scored based on ORR, there was notable variation in the definitions of objective response in these studies. Two studies used Radiological Assessment in Neuro-Oncology (RANO) criteria.^30,37^ The other studies used various definitions of response based on 2-dimensional or volumetric imaging. In 1 study, response criteria were amended during the study resulting in incorporation of stable disease (SD) into the definition of tumor response. The same study was not scorable as neither duration of response nor median progression-free survival (PFS) were reported.^36^ One study reported the results of BRAFV600E-targeted therapy in 2 study cohorts of a basket trial. For this analysis, these 2 study cohorts were treated as if they were separate single-arm studies.^30^ Another study represented a post hoc pooled efficacy analysis of various single-arm studies. Since the treatment indication, schedule of therapy, and response assessment criteria were the same across the studies included, this pooled efficacy analysis was deemed assessable.^37^

**Table 2.** ESMO MCBS 1.1 Assessment of Select Contemporary Single-Arm Clinical Studies Evaluating Novel Therapies for CNS Malignancies

Treatment indication/disease	Study name [Reference]	Intervention	No of subjects	1° endpoint	mPFS	mOS	ORR	Mean duration of response	Downgrade one level for ≥30% of pt. with grade 3/4 toxicities?	Upgrade 1 level for significant QoL improvement?	ESMO-MCBS score, Form 3^a^	Neuro-MCBS score
Subgroup, if applicable												
*BRAFV600E* mutant low-grade and high-grade glioma, recurrent, measurable disease	ROAR [30]	Dabrafenib + Trametinib		ORR (PR+CR+MR), per RANO								
High-grade glioma cohort			45		4.5 months	17.6 months	31%	13.6 months	yes	n/a^b^	2	2
Low-grade glioma cohort			13		14.0 months	Not reached	69%	27.5 months	no	n/a^b^	3	3
Papillary craniopharyngioma, *BRAFV600E* mutant, no prior radiation, measurable disease	Alliance 071601 [31]	Vemurafenib + Cobimetinib	16	ORR (CR+PR)	(-)	(-)	94%	(-)	yes	n/a^b^	2	2
Subependymal giant-cell astrocytomas in tuberous sclerosis, progressive	NCT00411619 [32], EXIST-1 [33]	Everolimus	28	Safety, efficacy (change in tumor volume within 6 months)	(-)	(-)	75%	(-)	yes	yes	3	3
Plexiform neurofibroma in NF1 patients, inoperable and measurable disease	NCT01362803 [34, 35]	Selumetinib	50	ORR (volumetric MRI)	(-)	(-)	68%	not reached	no	yes	4	4
Sporadic optic pathway and hypothalamic low-grade glioma without NF1, recurrent/progressive	PBTC-029 [36]	Selumetinib	25	ORR (CR+PR) sustained for ≥ 8 weeks	(-)	(-)	24% (*n* = 2 with MR)	(-)	no	n/a^b^	n/a^c^	n/a^c^
*H3K27M* mutant diffuse midline glioma, progressive disease with measurable disease at baseline.	Arrillaga-Romany et al. [37]	ONC201 (Dordaviprone)	50	ORR (CR+PR), per RANO	(-)	13.7 months	20%	11.2 months	no	n/a^b^	3	3

Abbreviations: CR = complete response; MR = minor response; n/a = not applicable; ORR = objective response rate; OS = overall survival; PFS = progression-free survival; PR = partial response; RANO = Response Assessment in Neuro-Oncology.

^a^Form 3: single-arm studies in "orphan diseases" and diseases with "high unmet clinical need", primary outcome PFS or ORR, scale 1–4.

^b^QoL studies not reported.

^c^DoR not reported

### Development of the Neuro-Oncology Magnitude of Clinical Benefit Scale

With the goal to make the evaluation of magnitude of clinical benefit more suitable to single-arm studies in neuro-oncology, we modified the ESMO-MCBS v1.1 Form 3 and developed the Neuro-Oncology Magnitude of Clinical Benefit Scale (Neuro-MCBS, Table 3).^8^ We proposed that an objective response assessment should be based on 2-dimensional tumor measurements and that Response Assessment Criteria in Neuro-Oncology (RANO) most applicable to the tumor type studied should be used, if available.^38,39^ Given that the RANO 2.0 criteria recognize minor response (25%–50% decrease in bidirectional tumor measurements) for non-enhancing tumors and given that durable SD represents a clinically meaningful outcome in neuro-oncological patients, we proposed to replace ORR with disease control rate (DCR) for the Neuro-MCBS. Consequently, duration of response (DoR) assessment was replaced by DCR. Based on these modifications, a preliminary score can be rendered on a 3-point scale. If there is significant reduction of toxicity and/or improvement in QoL, 1 additional point can be given for a maximal final Neuro-MCBS score of 4.

**Table 3.** Neuro-Oncology Magnitude of Clinical Benefit Scale (Neuro-MCBS) for single-arm studies

Step 1:	
Grade	Criteria
**3**	PFS ≥ 6 months or
	ORR (CR + PR + MR) ≥ 60% or
	DCR (CR + PR + MR + SD) ≥ 20 to < 60% and DoCB ≥ 9 months
**2**	PFS ≥ 3 to < 6 months or
	ORR (CR + PR + MR) ≥ 40 to < 60% or
	DCR (CR + PR + MR + SD) ≥ 20 to < 40% and DoCB ≥ 6 to < 9 months
**1**	PFS ≥ 2 to < 3 months or
	ORR (CR + PR + MR) ≥ 20 to < 40% and DoCB < 6 months
	DCR (CR + PR + MR + SD) ≥ 10 to < 20% and DoCB ≥ 6 months
**Step 2:**	
Upgrade 1 level if	QoL evaluated as secondary outcome and objectively improved or
	confirmatory, adequately sized, phase 4 experience available
	
Downgrade 1 level if	≥30% grade 3–4 toxicities impacting daily well-being, (not including myelosuppression, asymptomatic lab abnormalities, . . . ).
**Step 3:**	
	Create sum from score obtained in Step 1 and 2.
	Categorical Scale 1–4, maximum score of 4.

PFS = Progression-Free Survival, ORR = Objective Response Rate, CR = Complete Response, PR = Partial Response, MR = Minor Response, DCR = Disease Control Rates, SD = Stable Disease, DoCB = Duration of Clinical Benefit, QoL = Quality of Life.

Response assessments based on bidirectional tumor measurements; Response Assessment in Neuro-Oncology (RANO) criteria most applicable to tumor type should be used.

### Application of Neuro-MCBS in single-arm studies in neuro-oncology.

**Figure 1.**
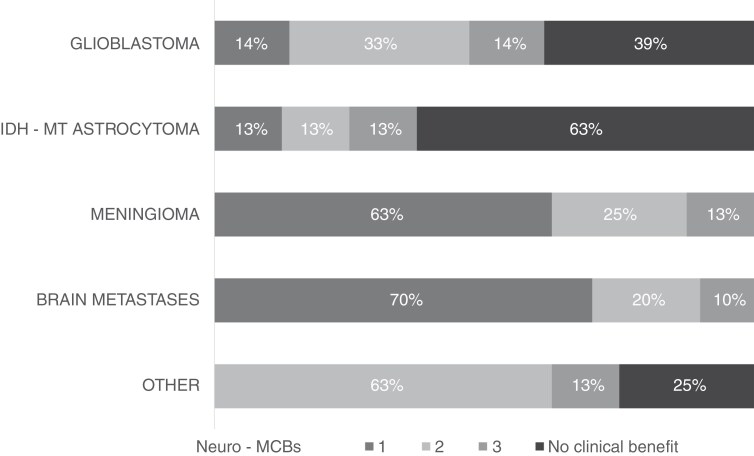
Application of Neuro-MCBS to select patient cohorts enrolled in MTB@ZPM.

As previously outlined, we reasoned that all neuro-oncology patients within MTB@ZPM could also be viewed as a series of single arms of a clinical study.^8^ To test application of the Neuro-MCBS, we applied this to individual evaluable patients receiving biomarker-guided targeted therapy as individual named patient protocols within the MTB@ZPM workflow. From February 1^st^, 2022, until January 31^st^, 2023, 70 patients were enrolled. All patients had received at least 1 line of prior therapy and had recovered at least 1 month from prior localized therapy (e.g. surgical resection or radiation) before beginning a targeted therapy. Among these patients, 5 of 8 patients with meningioma (63%) and 7 of 10 patients with brain metastases (70%) achieved a Neuro-MCBS score of 3, thereby considered to have derived a net clinical benefit from targeted therapy. Only 5 of 36 glioblastoma patients (14%) and one of 8 IDH-mutant astrocytoma patients (13%) achieved a Neuro-MCBS score of 3 (Figure 1, Table 4).

**Table 4.** Targeted therapies used in select patient cohorts enrolled in MTB@ZPM

Targeted therapy	*n*
EGFR inhibition	17
PD-1/PD-L1 inhibition	13
NTRK inhibition	2
PARP inhibition	3
TK inhibition	7
Multikinase inhibition	1
FGFR inhibition	4
mTOR inhibition	14
VEGF inhibition	2
IDH inhibition	5
CDK 4/6 inhibition	3

## Discussion

Herein, we assessed the magnitude of clinical benefit derived from various therapies for CNS malignancies when considering the associated toxicity by application of the ESMO-MCBS v1.1 and the ASCO-NHB v2 scale to 16 clinical studies evaluating systemic therapies for CNS malignancies. Of 10 therapies, 5 evaluated in comparative phase 3 clinical studies achieved an ESMO-MCBS score of 4, indicating that a net clinical benefit may be derived from these treatments. Four of 6 therapies evaluated in single-arm clinical studies, achieved an ESMO-MCBS score ≥ 3. To make the application of the ESMO-MCBS and ASCO-NHB more suitable to clinical studies in neuro-oncology, we developed the Neuro-MCBS, incorporating bidirectional tumor measurements by RANO criteria and DCR and DoCR assessments. The newly developed Neuro-MCBS was tested on 16 clinical studies and select patient cohorts enrolled in MTB@ZPM evaluating molecularly based therapies for advanced CNS malignancies. Molecularly based therapies were determined to be useful in the majority of patients with brain metastases and meningiomas, but less so in patients with gliomas.

This ESMO-MCBS v1.1 scale and ASCO-NHB v2 framework were developed to objectively assess the magnitude of clinical benefit of a novel oncologic therapy in the face of associated toxicity and effects on QoL. To the best of our knowledge, this is the first time that these frameworks are applied to clinical studies in CNS malignancies. Similar to prior reports of the application of the ESMO-MCBS v1.1 in other oncological settings,^40^ we identified several limitations of Forms 2a-c and Form 3 when it comes to neuro-oncology studies: (1) Non-inferiority studies can only be assessed by Form 2c if there is report on statistical non-inferiority *and* report of significantly reduced toxicity and/or improvement in QoL. In contrast to study assessments using Forms 2a and 2b, in which assessment and improvement of toxicity and QoL lead to “bonus points” and may increase the preliminary score, clinical studies assessed by Form 2c are precluded, i.e. punished if they cannot demonstrate improvement in toxicity and QoL. (2) In studies using ORR as the primary outcome measure (Form 2c or Form 3), there is limited guidance on how to assess ORR and various criteria are being used in (neuro-)oncological practice, including but not limited to RECIST v1.1 and various iterations of the RANO criteria. (3) Durable disease control, i.e. SD and minor responses may be considered clinically meaningful and reduce patient and caregiver distress. However, assessment of DCR and DoCB are not considered or credited in the ESMO-MCBS v1.1. The application of the ASCO value framework for “advanced diseases” to comparative clinical studies in neuro-oncology had similar limitations and led to highly variable results in the clinical studies assessed.

To address some of these shortcomings and to make magnitude of clinical benefit assessments more suitable to neuro-oncology studies, we developed the Neuro-MCBS.^8^ The Neuro-MCBS can be applied to single-arm studies and mandates objective response assessments to be based on bidirectional tumor measurements according to RANO criteria. It also incorporates assessment of disease stability, by including the evaluation of DCR and DoCB. We applied the Neuro-MCBS scale to the same 6 single-arm studies evaluated by the ESMO-MCBS v1.1 and applied it to the various CNS tumor cohorts enrolled in MTB@ZPM from February 1^st^, 2022, until January 31^st^, 2023. MTB@ZPM is a prospective single-center single-arm clinical study that aims at molecular-guided stratification and therapy of patients with advanced tumor diseases. We found that application of the Neuro-MCBS scale in both settings was feasible and resulted in valid assessments of the net clinical benefit derived from targeted therapies.

Our study has several limitations, e.g. the retrospective design and the limited number of available neuro-oncology studies demonstrating statistical superiority to which the ESMO-MCBS v1.1, ASCO-NHB v2 and Neuro-MCBS can be applied. While Forms 2a-c of the ESMO-MCBS scale and the ASCO-NHB framework were developed for assessment of comparative phase 3 studies only, most therapies used and trialed in neuro-oncology have not been evaluated in this setting. By refining Form 3 of the ESMO-MCBS vs1.1 and making this more applicable to neuro-oncology clinical studies, the Neuro-MCBS can be applied to single-arm phase 1 and 2 studies and, es demonstrated here, to even smaller case series and treatment cohorts as in MTB@ZPM. The ESMO-MCBS and Neuro-MCBS were developed for application to a broad spectrum of neuro-oncological disease entities and the criteria used to build a Neuro-MCBS score, e.g. PFS duration, ORR, and DCR, were chosen because they are representative indicators of clinical benefit across the neuro-oncological disease spectrum.

However, it is recognized and must be emphasized that the strongest level of evidence remains to be derived from sufficiently powered, prospective and comparative clinical studies. Nevertheless, the Neuro-MCBS represents a practical tool to clinicians who must gauge the potential clinical benefit of a novel therapy based on lower-level evidence from the limited available literature.

The valid assessment of clinical trial results by application of the ESMO-MCBS v1.1, ASCO-NHB v2, and the Neuro-MCBS is dependent on high-quality and detailed data reporting. The present study uncovered and confirmed important opportunities for improvement in the design and, particularly, in the reporting of future clinical studies in neuro-oncology. This includes increased depth and breadths of toxicity reporting, improved presentation of long-term follow-up data, and better incorporation and reporting of patient-reported outcomes, such as self-reported health-related QoL. While objective QoL assessments in clinical trials are resource-intense for patients and investigators, these assessments offer important insight into tolerability of a neuro-oncological therapy and inform discussions around clinical benefit (“is this therapy worth it”) of a neuro-oncological therapy. In addition, more unified radiographic assessment criteria underling objective response evaluations are necessary to minimize the potential source for errors and bias when assessing the efficacy of medical therapies.

By incorporating and addressing some of the shortcomings identified in the ESMO-MCBS v1.1 Form 3, we developed the Neuro-MCBS as a practicable tool for neuro-oncologists for an objective and valid assessment of the clinical benefit derived from therapies in neuro-oncology. However, further field-testing and validation of the Neuro-MCBS in a larger set of contemporary clinical studies will be necessary. Moreover, more rigorous reporting not only of outcome data but also of toxicity and QoL results from clinical studies conducted in neuro-oncology are imperative for full application of the Neuro-MCBS and to allow for a truly objective assessment of these therapies.

## Conclusions

The ESMO-MCBS v1.1 and ASCO-NHB v2 represent valid and objective assessment tools to gauge the impact of an oncological therapy considering the associated toxicities but have important limitations when applied to neuro-oncology studies. The Neuro-MCBS established herein is a comprehensive and clinically relevant assessment tool that was applied to 10 comparative phase 3 and 6 single-arm phase 1 studies. The Neuro-MCBS is particularly suitable to neuro-oncology studies and incorporates clinical, imaging, and survival outcome data to determine the degree of clinical benefit from targeted therapies in patients with primary and secondary CNS tumors. Its incorporation into neuro-oncology practice may result in practical and objective evaluation of the net clinical benefit derived from a new therapy and may provide useful guidance to patients and physicians when choosing (or not choosing) the next line of treatment.

## Data Availability

The data presented in this manuscript will be made available upon reasonable request.
